# Hyperreactivity of Salivary Alpha-Amylase to Acute Psychosocial Stress and Norepinephrine Infusion in Essential Hypertension

**DOI:** 10.3390/biomedicines10071762

**Published:** 2022-07-21

**Authors:** Lisa-Marie Walther, Roland von Känel, Claudia Zuccarella-Hackl, Petra H. Wirtz

**Affiliations:** 1 Biological Work and Health Psychology, Department of Psychology, University of Konstanz, 78464 Konstanz, Germany; lisa-marie.walther@uni-konstanz.de; 2 Centre for the Advanced Study of Collective Behaviour, University of Konstanz, 78464 Konstanz, Germany; 3 Department of Consultation-Liaison Psychiatry and Psychosomatic Medicine, University Hospital Zurich, University of Zurich, 8091 Zurich, Switzerland; roland.vonkaenel@usz.ch (R.v.K.); claudia.hackl-zuccarella@usz.ch (C.Z.-H.)

**Keywords:** essential hypertension, acute psychosocial stress, Trier Social Stress Test, physiological hyperreactivity, salivary alpha-amylase, norepinephrine infusion

## Abstract

It is unknown whether the observed general physiological hyperreactivity to acute psychosocial stress in essential hypertension also extends to salivary alpha-amylase (sAA), a surrogate sympathetic nervous system marker. Here, we investigated sAA reactivity to acute psychosocial stress in essential hypertensive males (HT) as compared to normotensive controls (NT). To shed light on underlying mechanisms, we moreover tested for sAA reactivity following a standardized norepinephrine (NE) infusion. We hypothesized that both acute psychosocial stress and an NE infusion of similar duration would lead to greater sAA reactivity in HT than in NT. In the *stress study*, we examined sAA reactivity to 15 min of acute psychosocial stress induced by the Trier Social Stress Test (TSST) in 19 HT and 23 NT up to 40 min after stress. In the *infusion study*, 20 HT and 22 NT received a standardized NE infusion (5 μg/mL/min) over 15 min mimicking NE release in reaction to acute psychosocial stress. HT exhibited greater sAA reactivity to the TSST as compared to NT (*p* = 0.049, η_p_^2^ = 0.08, *f* = 0.29). In reaction to the standardized NE infusion, HT showed higher sAA reactivity as compared to NT (*p* = 0.033, η_p_^2^ = 1.00, *f* = 0.33). Our findings suggest stress-induced sAA hyperreactivity in essential hypertension that seems to be at least in part mediated by a higher reactivity to a standardized amount of NE in HT. With respect to clinical implications, sAA stress reactivity may serve as a noninvasive marker indicative of early cardiovascular risk.

## 1. Introduction

Essential hypertension (EHT), the chronic elevation of blood pressure (BP) without secondary causes, is a major risk factor for the development of cardiovascular diseases [1]. In this context, the observed generalized physiological hyperreactivity to acute (psychosocial) stress in EHT has been proposed to play a mechanistic role [2,3,4]. More precisely, in response to acute psychosocial stress, hypertensive individuals (HT) have been shown to exhibit enhanced reactivity across several physiological systems including the sympathetic–adrenomedullary (SAM) axis [4,5,6,7,8,9,10]. Within the last two decades, salivary alpha-amylase (sAA) has emerged as a surrogate, noninvasive biomarker for sympathetic nervous system (SNS) activity [11]. The enzyme sAA is primarily produced in the salivary glands and involved in the digestion of macromolecules (e.g., starches) [12]. Accumulating evidence indicates that sAA release is stimulated by acute SNS activation, in terms of both psychological and physiological (e.g., treadmill, running, or bicycle exercise) stress (for review see: [11]). Moreover, increased sAA activity was observed after pharmacologically induced sympathetic activation by the infusion of norepinephrine (NE) [13,14] or other substances [15,16].

In EHT, sAA activity has hitherto only been investigated under basal, i.e., nonstress, conditions. Here, HT showed enhanced sAA activity over the course of the day [17] as well as in the evening [18] as compared to normotensive controls (NT). Moreover, antihypertensive medication to lower BP and sympathetic overactivity in HT was associated with lower circadian sAA activity [17]. Similarly, transient increases in morning sAA in HT during withdrawal of antihypertensives were reversed with reinstallation of antihypertensive pharmacotherapy [19]. So far, sAA reactivity in response to acute stress has not been investigated in EHT, neither in reaction to psychological or physiological stress nor to pharmacological stimulation. Given the observed enhanced sAA activity in HT under resting conditions [17,18] and the observed SNS hyperreactivity to acute psychosocial stress in EHT [9,10], one would expect sAA reactivity in response acute psychosocial stress induction to be increased in EHT as compared to NT.

What mechanisms may underlie an expected sAA hyperreactivity to stress in EHT? sAA stress reactivity has been proposed to reflect increases in catecholamines, in particular of NE, induced by activation of the SNS [20,21]. Indeed, response patterns in reaction to acute stress are similar for sAA and the plasma catecholamines NE and epinephrine in NT, with an immediate increase at the onset of stress and fast recovery after stress cessation [22,23]. Most studies assessing associations between plasma catecholamine reactivity and sAA reactivity to acute mental stress in NT report significant associations in particular with NE [20,22,23,24,25], but not uniformly so [21,26]. Indeed, we previously found evidence for a mediating role of NE in sAA increases: a stress reactivity-mimicking NE infusion induced substantial sAA increases in NT with similar kinetics as observed in reaction to acute stress, i.e., immediate sAA increases with infusion onset and fast recovery after the end of the infusion [13]. This suggests that the mechanisms underlying stress-induced sAA increases may involve NE.

HT showed elevated basal plasma NE levels [7,9], and some studies report enhanced stress-induced plasma NE increases in HT compared to NT [9,27]. Given this and the previously described associations between NE increases and sAA reactivity, higher stress-induced NE increases in EHT may represent a mechanism underlying the expected sAA hyperreactivity to acute stress in EHT. However, not all studies support associations between NE increases and concomitant sAA increases [21,26], and NE stress reactivity is not consistently increased in HT as compared to NT [7,28]. Moreover, we recently found exaggerated cardiovascular reactivity in EHT as compared to NT to the same dosage of infused NE [29]. Thus, the expected sAA hyperreactivity to stress in EHT might alternatively, in addition to or instead of a proportional reactivity to stress-induced NE release, result from a higher reactivity to a standardized amount of NE. So far, associations between stress-induced NE and sAA increases have been investigated in NT only. Moreover, sAA reactivity to (a standardized) NE infusion has not yet been investigated in EHT.

Here, we investigated for the first time sAA reactivity to acute psychosocial stress in essential hypertensive males as compared to NT. We expected sAA reactivity in response to acute psychosocial stress to be increased in HT as compared to NT. Moreover, to shed light on the mechanisms underlying the expected sAA hyperreactivity to acute psychosocial stress in EHT, we tested for proportional linear associations between NE and sAA reactivity to acute psychosocial stress as well as for an enhanced reactivity to infusion of a standardized amount of NE that mimics NE release in reaction to acute psychosocial stress. Based on our previous cardiovascular results [29], we hypothesized sAA increases following NE infusion to be higher in HT as compared to NT.

## 2. Materials and Methods

Here, we present data from two studies, an acute psychosocial stress study applying the Trier Social Stress Test (stress study) and an infusion study applying a stress reactivity-mimicking NE infusion of identical length (NE infusion study). Both studies were formally approved by the respective ethics committees (stress study: Ethics Committee of the State of Zurich, Switzerland; NE infusion study: Ethics Committee of the State of Bern, Switzerland) and conducted in accordance with the Declaration of Helsinki principles. All participants provided written informed consent prior to participation.

### 2.1. Acute Psychosocial Stress Study

#### 2.1.1. Study Participants and Assessment of Essential Hypertension

This first study is part of a larger project investigating stress reactivity in EHT [7,8,30]. As previously described in detail, we recruited hypertensive and normotensive otherwise healthy, nonsmoking, and medication-free males [7]. Hypertension status was assessed by three seated screening BP measurements. Based on the resulting average screening BP participants were categorized into HT and NT following the World Health Organization (WHO)/International Society of Hypertension (ISH) definition (hypertension: SBP ≥ 140 mmHg and/or DBP ≥ 90 mmHg) [31]. Please see Appendix A for more details regarding EHT assessment. The initial sample of 22 HT and 26 NT was reduced to 19 HT and 23 NT because of missing sAA data. More precisely, due to the higher prioritization of salivary cortisol in biochemical analysis [7], there was not sufficient volume of saliva left for sAA analyses.

#### 2.1.2. Procedure and Psychosocial Stress Induction

Psychosocial stress was induced by the Trier Social Stress Test (TSST) [32] comprising a 5 min introduction and preparation phase, followed by a 5 min mock job interview and a 5 min mental arithmetic task. The procedure took place in a standing position in front of an audience with video and audio recording. The TSST reliably elicits stress responses in various physiological parameters, including sAA [21]. In order to control for circadian fluctuations on stress hormones, experimental sessions started between 14:00 h and 16:00 h. Participants abstained from physical exercise, alcohol, and caffeinated beverage since the previous evening and from food and drink (except for water) for 2 h before the start the of the experimental session. The stress procedure started after a 45 min acclimation phase, and participants remained seated in a quiet room for another 60 min after task completion. For the investigation of sAA reactivity in reaction to the TSST, we considered five saliva samples collected by chewing on cotton rolls for 1 min: one baseline sample obtained 1 min before TSST introduction as well as four samples obtained +1, +10, +20, and +40 min after TSST cessation. To test whether sAA increases would relate to NE increases, we assessed NE plasma levels at baseline, i.e., 1 min before TSST introduction, and peak, i.e., +1 min after TSST cessation. The NE assessment has been previously described in detail [7].

### 2.2. Norepinephrine Infusion Study

#### 2.2.1. Study Participants and Assessment of Essential Hypertension

This second study is part of a larger project investigating the effects of an NE stress reactivity-mimicking NE infusion [13,29,33,34]. As previously described in detail, we recruited hypertensive and normotensive otherwise healthy and medication-free males between 30 and 66 years [29]. To assess hypertension status, we applied a two-step procedure comprising home and screening BP measurements. Categorization in HT and NT was based on the European Society of Hypertension recommendations for home BP measurements (hypertension: average home SBP ≥ 135 mmHg and/or average home DBP ≥ 85 mmHg) [35] and the WHO/ISH definition for study or ambulatory BP measurements (hypertension: SBP ≥ 140 mmHg and/or DBP ≥ 90 mmHg) [31]. For more details regarding EHT assessment, please see Appendix A. Due to the priority analysis of salivary cortisol, our initial sample of 24 HT and 24 NT was reduced to 20 HT and 22 NT with enough sample material left for sAA analyses.

#### 2.2.2. Procedure and Norepinephrine Infusion

In a single-blind, placebo-controlled, within-subject design, all participants completed three experimental trials on three separate days with two sequential standardized infusions. Experimental trials varied in the combination of infused substances and trial order was randomized using a Latin Square Design as previously described in detail [29]. For the present research question, only one trial is of relevance where we tested the effects of a NE stress reactivity-mimicking NE infusion. In that trial, a 1 min saline (Sal) infusion (infusion 1) was followed by a 15 min NE infusion (infusion 2) with 5 min time between the two infusions. NE (Sintetica SA, Mendrisio, Switzerland) was diluted in Sal, and the resulting solution of 5 μg/mL was infused with a constant speed of 1 mL/min over a 15 min period (rendering a total of 75 μg NE). We chose this dosage based on earlier studies showing that a dose of 5 μg/mL/min NE, yielding plasma levels in excess of 1800 pg/mL, is required to produce BP increases comparable to acute (mental) stress; we could confirm this previously [13,29]. The 15 min infusion time interval was chosen based on the duration of the TSST. Thereby, our NE infusion mimicked the duration and effectiveness of NE release in reaction to psychosocial stress induced by the TSST with respect to BP increases.

Similar to the stress study, participants abstained from alcohol and caffeinated beverages since the previous evening. Moreover, they abstained from physical activity for 24 h before study participation and kept a regular sleep–wake rhythm the night before participation, with sleep starting between 22:30 h and 24:00 h and awakening between 07:00 h and 09:00 h. Participants reported to the laboratory at 11:45 h, where they received a standardized meal. The experimental procedure started at 13:00 h. In a 10 min introduction phase, the testing procedure was explained. Catheter insertion into the brachial vein of the dominant arm for the infusions followed. After a further 45 min acclimatization phase, the infusion phase started with the 1 min Sal infusion. Following a 5 min waiting period, NE was infused for 15 min. Participants were in supine position lying on a bed for the entire experimental procedure.

For the investigation of sAA reactivity to NE infusion, we considered four saliva samples collected by chewing on cotton rolls for 1 min: one baseline sample obtained immediately before beginning of the infusion phase and three samples obtained +1, +10, and +20 min after the end of the NE infusion. To test for successful NE infusion, we assessed plasma NE levels at baseline, i.e., 1 min before the NE infusion started and +1 min after the end of the NE infusion. The NE assessment has previously been described in detail [29].

### 2.3. Biochemical Analyses

We applied the same biochemical analyses in both studies.

#### 2.3.1. Salivary Alpha-Amylase

For the assessment of alpha-amylase, saliva samples were collected using Salivettes (Sarstedt, Rommelsdorf, Germany) and stored at −20 °C until analysis. Thawed saliva samples were centrifugated at 3000 rpm for 10 min, yielding low-viscosity saliva. For determination of sAA, we used an enzymatic colorimetric assay (Roche 11555685 Alpha-Amylase Liquid acc, Roche Diagnostics GmbH, Rotkreuz, Switzerland) and automatic analyzers (stress study: Cobas Mira, Roche Diagnostics GmbH, Rotkreuz, Switzerland; NE infusion study: Synergy H1, BioTek Instruments GmbH, Bad Friedrichshall, Germany) following methodological recommendations [36]. Amylase activity was expressed in units per milliliter (U/mL). Intra-assay variation was 1.9%, and inter-assay variation was 7.4%.

#### 2.3.2. Plasma Norepinephrine

For the assessment of plasma NE, venous blood was drawn into ethylenediaminetetraacetic acid (EDTA)-coated monovettes (Sarstedt, Numbrecht, Germany). After centrifugation at 2000 g and 4 °C for 10 min, obtained plasma was immediately aliquoted in polypropylene tubes and stored at −80 °C until analysis. Plasma NE levels were determined using high-pressure liquid chromatography with inter- and intra-assay variances < 5% and a lower detection limit of 0.25 pg/mL (Laboratory for Stress Monitoring, Göttingen, Germany [37]). For the NE infusion study, NE data of one NT participant were missing because of technical problems with high-pressure liquid chromatography. Biochemical analyses were performed shortly after the end of data collection of each study.

### 2.4. Statistical Analyses

Statistical analyses were performed using SPSS statistical software package for Macintosh (Version 28.0, IBM SPSS Statistics, Chicago, Il, USA). Data are presented as mean ± standard error of the mean (SEM). All analyses were two-tailed with level of significance set at *p* < 0.05 and p-values < 0.10 interpreted as marginally significant. Body mass index (BMI) was calculated by the formula BMI = body weight (kg)/body height squared (m^2^). Mean arterial pressure (MAP) was calculated as follows: MAP = 2/3*screening DBP + 1/3* screening SBP. A priori power analysis revealed that with respect to the main analyses of interest, the lowest sample size of *n* = 42 allowed detection of group-by-time interactions with *f* = 0.15 (representing small- to medium-sized effects) with a power of (1 − β) = 0.80 or greater, α = 0.05 and an expected observed average correlation of repeated measures of *r* > 0.62. With respect to linear regression analyses between NE increases and sAA reactivity in the stress study, the sample size of *n* = 42 allowed detection of medium-sized effects of *R*^2^ = 0.16 given a power of (1 − β) = 0.80 or greater and α = 0.05.

The following data analysis procedure was applied for both studies:

To test for differences in participants’ characteristics including sAA and NE concentrations at baseline, we calculated univariate analyses of variance (ANOVAs).

To test for differences in sAA reactivity to the TSST and to the NE infusion between HT and NT, we first calculated absolute sAA changes from baseline following previous methodological recommendations regarding sAA analyses [36]. We then performed repeated measures univariate analyses of covariance (ANCOVAs) with group (HT vs. NT) as the independent variable and repeated sAA changes as repeated dependent variables. Due to the potentially confounding effects of age and BMI on sAA (re)activity [38,39], we controlled for age and BMI as covariates in our repeated measures ANCOVAs. In the NE infusion study, we also controlled for trial order as an additional covariate to account for potential sequence effects. Post hoc testing comprised (1) univariate ANOVAs comparing sAA level changes from baseline between HT and NT at each measurement time point and (2) separate analyses in HT and in NT with repeated measures ANOVAs (2a) considering all measurement time points and (2b) between baseline and every later measurement time point separately.

In the stress study, we moreover tested for associations between TSST-induced sAA reactivity and plasma NE increases. Considering all participants, we calculated (multiple) linear regression analyses with either sAA reactivity (area under the curve with respect to increase (AUCi) [40]); sAA maximum increases (difference between baseline and peak, i.e., +1 min after the TSST); or sAA peak levels (i.e., +1 min after the TSST) as the dependent variable. As the independent variable we considered plasma NE increases (difference between baseline and peak, i.e., +1 min after TSST) or plasma NE peak levels (i.e., +1 min after the TSST). We performed all regression analyses with and without controlling for age and BMI. We repeated the (multiple) linear regression analyses in NT and HT separately.

To verify successful manipulation and to identify potential group differences in plasma NE reactivity to NE infusion, respectively, we calculated a repeated measures ANCOVA with group (HT vs. NT) as the independent variable and repeated plasma NE levels as the repeated dependent variable. Trial order was controlled.

Preceding statistical analyses, data were tested for normal distribution (Levene test) and homogeneity of variance (Kolmogorov–Smirnov test). To protect against violations of sphericity, we applied Huynh–Feldt correction for the degrees of freedom where appropriate.

Effect size parameters (*f*) were calculated from partial eta squared (η_p_^2^) using G*Power for Macintosh (Version 3.1.9.6; Heinrich Heine Universität, Düsseldorf, Germany) and are reported where appropriate (effect size conventions: *f* 0.10 = small, 0.25 = medium, 0.40 = large).

## 3. Results

### 3.1. Participants’ Characteristics

Table 1 depicts the participant characteristics for the stress study and the NE infusion study. As expected, HT had significantly higher (home and/or screening) SBP and DBP (*p*’s ≤ 0.001) as well as higher NE baseline levels (*p*’s ≤ 0.018) as compared to NT in both studies. We observed significantly higher sAA baseline levels in HT (*p* = 0.047) in the NE infusion study but borderline significantly higher sAA baseline levels in NT in the stress study (*p* = 0.077). There were no differences in terms of age and BMI between NT and HT in both studies (*p*’s ≥ 0.11).

### 3.2. Salivary Alpha-Amylase and Norepinephrine Reactivity in Response to Acute Psychosocial Stress

#### 3.2.1. Salivary Alpha-Amylase

**Figure 1:**
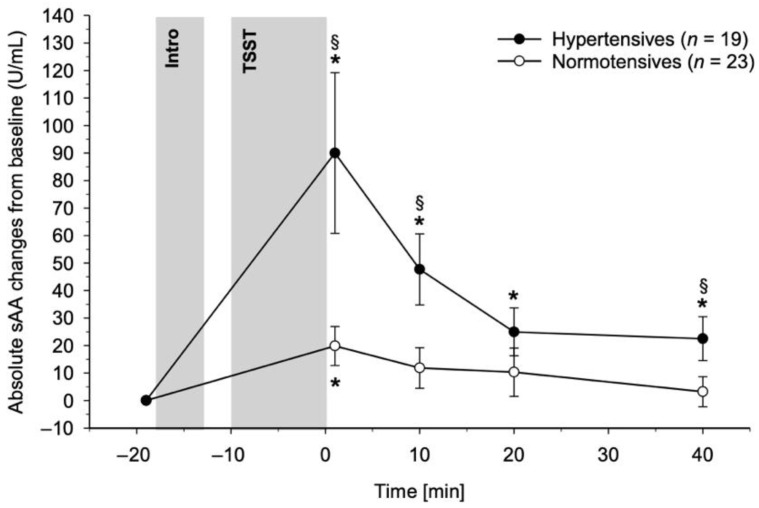
Stress study. Salivary alpha-amylase (sAA) reactivity to acute psychosocial stress in hypertensive individuals (black dots) and normotensive controls (white dots). Repeated measures ANCOVA revealed that hypertensive individuals showed higher sAA stress reactivity as compared to normotensive controls (*p* = 0.049). TSST = Trier Social Stress Test. Paragraphs (§) indicate significant differences between hypertensive and normotensive participants. Asterisks (*) indicate significant differences from baseline within the respective group.

HT and NT differed in their sAA reactivity to the TSST (interaction group-by-time: *F*(1.71, 64.90) = 3.33, *p* = 0.049, η_p_^2^ = 0.08, *f* = 0.29) with HT showing greater sAA increases compared to NT (Figure 1). Post hoc testing comparing sAA level changes from baseline between HT and NT at each measurement time point revealed significantly higher sAA level increases in HT as compared to NT +1 min and +10 min after stress cessation (*p*’s ≤ 0.016) as well as +40 min after stress cessation (*p* = 0.047). No significant group differences were observed +20 min after stress cessation (*p* = 0.25). Further post hoc testing repeating analyses separately in each group showed that both HT and NT exhibited significant sAA level increases in reaction to stress (main effect of time: NT: *F*(3.18, 70.03) = 2.74, *p* = 0.047, η_p_^2^ = 0.11, *f* = 0.35; HT: *F*(1.35, 24.26) = 5.95, *p* = 0.015, η_p_^2^ = 0.25, *f* = 0.57). While NT showed significantly increased sAA levels as compared to baseline immediately after stress (*p* = 0.010) but not later (*p*’s ≥ 0.12), we observed significantly increased sAA levels at all measurement time points after stress in HT (*p*’s ≤ 0.011).

#### 3.2.2. Norepinephrine

Notably, in the current sample with complete sAA data, we could confirm our previous NE results [7]: over all, NE levels significantly increased following stress (main effect of time: *F*(1, 40) = 124.79, *p* < 0.001, η_p_^2^ = 0.76, *f* = 1.77) but without differences between HT and NT (interaction group-by-time: *F*(1, 40) = 0.13, *p* = 0.72) with HT showing higher NE levels before and after stress (main effect of group: *F*(1, 40) = 4.43, *p* = 0.042, η_p_^2^ = 0.10, *f* = 0.33).

#### 3.2.3. Associations between Salivary Alpha-Amylase and Norepinephrine

We additionally tested for associations between sAA reactivity and stress-induced plasma NE increases. Plasma NE levels immediately after stress predicted sAA levels immediately after stress with marginal significance without (β = 0.276, *R*^2^ = 0.076, *p* = 0.077) but not after controlling for age and BMI (*p* = 0.26). Examining HT and NT separately, plasma NE levels immediately after stress predicted sAA levels immediately after stress in NT (without covariates: β = 0.380, *R*^2^ = 0.145, *p* = 0.073; with covariates: *p* = 0.37) but not in HT (*p*’s ≥ 0.40). Notably, there were no associations between stress-induced plasma NE increases and either aggregated sAA reactivity (AUCi: *p*’s ≥ 0.63) or sAA maximum increases (*p*’s ≥ 0.58) with or without controlling for covariates in all participants. There were no associations, either in NT or in HT, between stress-induced plasma NE increases and either sAA reactivity (AUCi: NT: *p*’s ≥ 0.44; HT: *p*’s ≥ 0.78) or sAA maximum increases (NT: *p*’s ≥ 0.62; HT: *p*’s ≥ 0.61) with or without controlling for covariates.

### 3.3. Salivary Alpha-Amylase and Norepinephrine Reactivity to Norepinephrine Infusion

#### 3.3.1. Norepinephrine

As described previously [13,29,33,34], successful NE infusion, i.e., increases in plasma NE levels in reaction to NE infusion, was also confirmed for the current study (main effect of time: *F*(1, 37) = 35.68, *p* < 0.001, η_p_^2^ = 0.49, *f* = 0.98). The groups did not differ in their plasma NE level increases in reaction to NE infusion (interaction group-by-time: *F*(1, 37) = 1.57, *p* = 0.22). Moreover, there was no main effect for group (*F*(1, 37) = 0.65, *p* = 0.43).

#### 3.3.2. Salivary Alpha-Amylase

**Figure 2:**
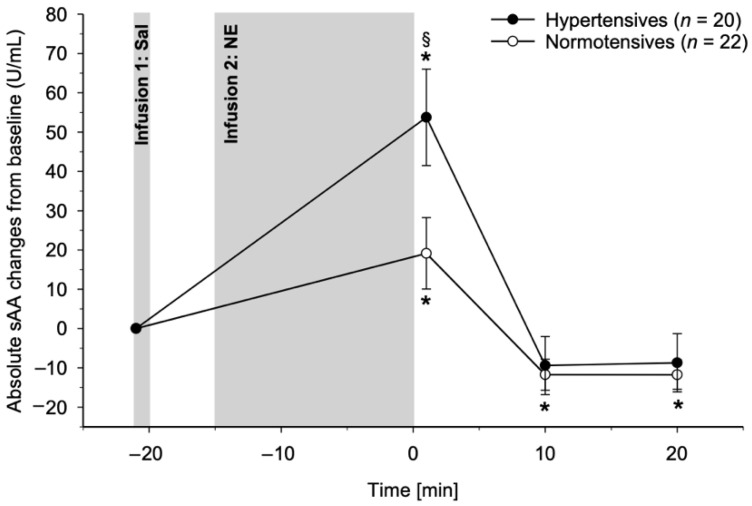
NE infusion study. Salivary alpha-amylase (sAA) reactivity to norepinephrine (NE) infusion with prior saline (Sal) infusion in hypertensive individuals (black dots) and normotensive controls (white dots). Repeated measures ANCOVA revealed that hypertensive individuals had higher sAA reactivity to NE infusion as compared to normotensive controls (*p* = 0.045). Paragraphs (§) indicate significant differences between hypertensive and normotensive participants. Asterisks (*) indicate significant differences from baseline within the respective group.

HT and NT differed in their sAA reactivity to NE infusion (interaction group-by-time: *F*(1.64, 59.17) = 3.89, *p* = 0.033, η_p_^2^ = 1.00, *f* = 0.33) with HT showing greater sAA increases compared to NT (Figure 2). Post hoc testing comparing sAA level increases from baseline between HT and NT at each post infusion measurement time point revealed significantly higher sAA level increases in HT immediately after NE infusion (*F*(1, 40) = 5.25, *p* = 0.027, η_p_^2^ = 0.12, *f* = 0.36) but not at later measurement time points (*p*’s ≥ 0.71). In post hoc analyses, this reactivity difference was further tested by repeating the analyses in each group separately: overall, the NE infusion induced significant increases in sAA levels in both HT and NT (main effect of time: NT: *F*(1.37, 28.78) = 8.78, *p* < 0.001, η_p_^2^ = 0.30, *f* = 0.65; HT: *F*(1.57, 29.78) = 16.72, *p* < 0.001, η_p_^2^ = 0.47, *f* = 0.94). While both groups showed significantly increased sAA levels as compared to baseline immediately after NE infusion (NT: *F*(1, 21) = 4.41, *p* = 0.048, η_p_^2^ = 0.17, *f* = 0.46; HT: *F*(1, 19) = 19.20, *p* < 0.001, η_p_^2^ = 0.50, *f* = 1.01), later obtained sAA levels (+10 and +20 min after the end of NE infusion) did not differ from baseline in HT (*p*’s ≥ 0.22) but were decreased as compared to baseline in NT (*p*’s ≤ 0.007).

## 4. Discussion

Here, we investigated for the first time sAA reactivity in response to acute psychosocial stress in essential hypertensive males as compared to NT. Moreover, to shed light on the mechanisms underlying the expected sAA hyperreactivity to acute psychosocial stress induction in EHT, we first tested for proportional linear associations between NE and sAA reactivity to acute psychosocial stress. Second, we tested whether the expected sAA hyperreactivity in EHT may result from an enhanced reactivity to a standardized amount of NE by applying a constant, not weight-adjusted NE infusion that mimics NE release in reaction to acute psychosocial stress.

Our first main finding is that unmedicated, otherwise healthy essential hypertensive males showed significantly greater sAA reactivity to acute psychosocial stress as compared to NT. Notably, group reactivity differences were most pronounced immediately after stress termination. The observed enhanced sAA stress reactivity in HT supports the widely accepted physiological hyperreactivity hypothesis to acute stress in HT [4].

With respect to the mechanisms underlying the observed expected sAA hyperreactivity to acute psychosocial stress in EHT, our second main finding is that plasma NE levels immediately after stress termination were associated with sAA activity immediately after stress termination in NT but not in HT. This relationship aligns with previous studies in NT showing significant associations between plasma NE and sAA after mental stress [20,22,23,24,25] and provides further evidence for sAA reactivity as an indicator for stress-induced sympathetic activation [11,23]. The current study was the first to investigate associations between plasma NE and sAA activity in reaction to stress in HT. Given that in our HT group, plasma NE was not associated with sAA following acute psychosocial stress, the sAA hyperreactivity to acute psychosocial stress in HT is unlikely to be attributed to the generally higher plasma NE levels in EHT [7,28].

As a third main finding, we observed significantly higher sAA reactivity to NE infusion in HT as compared to NT, with most pronounced group differences immediately after the end of NE infusion. In other words, our HT reacted more strongly to a standardized amount of NE. Together with the findings of the stress study, this observation may explain on the one hand why we could not find an association between plasma NE and sAA after acute psychosocial stress in our HT group. On the other hand, it implies that the observed sAA hyperreactivity to acute stress in EHT may result from a higher reactivity to a standardized amount of NE. Notably, it remains unclear from our data whether the observed higher reactivity to NE in HT may occur in addition to or instead of a proportional linear reactivity to plasma NE levels, including stress-induced NE release. Notably, as cardiovascular stress reactivity is primarily mediated by catecholamines [41], our sAA results concur with our recently reported cardiovascular hyperreactivity in EHT as compared to NT to the same dosage of infused NE [29].

What mechanisms may underlie the observed higher reactivity to NE infusion in our HT? As NE infusion-induced sAA increases are supposedly mediated by β-adrenergic receptors [13,14,19,42], we speculate that alterations in β-adrenergic receptor functioning, either in terms of hypersensitization or increased receptor density, may account for the enhanced sAA reactivity to a standardized amount of NE in HT. So far, differences between HT and NT in functioning or density of β-adrenergic receptors located in the salivary gland have not been investigated. Moreover, conclusions from studies on cardiac, vascular, renal, or lymphocyte β-adrenergic receptor functioning or density would be misleading as receptor alterations are highly tissue-specific [43]. Apart from adrenergic receptor functioning, one could assume that salivary flow rate may play a role. However, this is unlikely, as most studies could not observe differences between HT and NT in salivary flow rate [18,44], and stress-induced increases in sAA have been shown to be independent of saliva flow rate [45]. Indeed, we assessed saliva flow rate in the stress study and could confirm that the groups did not differ in salivary flow rate and that controlling for salivary flow rate did not change sAA results (see Appendix B).

Given that we found sAA stress reactivity differences between an at-risk population and a healthy population, clinical implications of our study include that sAA reactivity may serve as an easy-to-collect measure indicative of early cardiovascular risk. Indeed, previous research revealed that sAA is among the protein biomarkers associated with new-onset of atherosclerotic cardiovascular disease [46]. Moreover, given the role of the sympathetic over-activity in potential consequences of hypertension progression [47] such as proteinuria and kidney disease [48,49,50] but also cardiac damage and future cardiovascular prognosis [51,52,53], sAA reactivity could also serve as risk indicator at later cardiovascular disease stages. Currently, salivary biomarkers, but not sAA, are increasingly employed in screening, diagnosis, and monitoring of kidney functioning [54]. Whether the additional consideration of sAA (re)activity in this context is beneficial remains to be elucidated.

Strengths of our study include, first, the combination of investigating stress reactivity effects with those of an infusion study designed to mimic duration and effectiveness (in terms of effects on BP) of NE release during acute psychosocial stress. This allows for the investigation of underlying mechanisms. Moreover, we used standardized experimental manipulations: (1) the valid and widely used TSST to induce psychosocial stress and (2) a standardized, not weight-adjusted NE infusion to prevent potential confounding of weight-adjusted dosage due to the higher weight in EHT [1]. Second, we followed state-of-the-art recommendations for sAA sampling, storage, measurement, and data analysis [17,36,55]. Third, we controlled for a variety of potential confounders during recruitment, conduction of the study, and in our statistical analyses. Our study also has its limitations. First, the generalizability of our findings is restricted to normotensive and medication-free essential hypertensive males. Furthermore, whether antihypertensive medication can attenuate the sAA hyperreactivity to stress remains to be investigated [17]. Second, as parasympathetic activity likewise is involved in sAA secretion [56], we cannot exclude that additive or interactive effects of parasympathetic and sympathetic activity affected our results despite our explicit chewing instruction that aimed at standardizing parasympathetic activity. Notably, parasympathetic activity has been proposed to be reduced in HT [57]. Third, alpha-amylase is not exclusively produced by the salivary glands but also by the pancreas [58]. Given that our measuring method detects both alpha-amylase from the salivary glands and from the pancreas, we cannot rule out potential confounding effects. However, due to the collection of saliva samples with salivettes, we consider potential confounding to be low. Forth, although we performed a comprehensive hypertension assessment procedure for both studies, we cannot completely exclude diagnosis of white coat or masked hypertension for participants of the stress study as they did not provide home BP recording. However, as screening BP measurements were taken in a nonclinical setting and as baseline study BP supported our screening BP data [7], we consider it unlikely that our hypertension classification in the stress study is biased due to white coat or masked hypertension. Last, given our relatively small sample size, especially when considering NT or HT separately, we cannot rule out that the stress study was underpowered to detect smaller effects in our regression analyses [59].

## 5. Conclusions

Taken together, our study reveals that the physiological hyperreactivity to stress in EHT also applies to the SNS surrogate marker sAA. Moreover, our findings suggest that an enhanced reactivity to a given amount of NE in HT as compared to NT may contribute to the observed sAA hyperreactivity to stress in HT.

## Figures and Tables

**Table 1:** Characteristics of normotensive (NT) and hypertensive (HT) participants in the stress study and the NE infusion study.

	Stress Study			NE Infusion Study		
	NT (*n* = 23)	HT (*n* = 23)	*p*	NT (*n* = 22)	HT (*n* = 20)	*p*
Age (years)	42.13 ± 2.87 (21–65)	46.74 ± 3.37 (22–64)	0.30	50.32 ± 2.13 (29–64)	54.50 ± 1.37 (40–64)	0.11
BMI (kg/m^2^)	25.55 ± 0.66 (20.66–34.33)	26.31 ± 0.47 (22.59–31.41)	0.37	24.48 ± 0.45 (21.68–29.04)	25.78 ± 0.67 (21.29–31.93)	0.11
Home SBP (mmHg)	-	-	-	*n* = 15 122.23 ± 1.79 (110.00–134.50)	*n* = 15 143.48 ± 2.48 (124.33–164.50)	**<0.001**
Home DBP (mmHg)	-	-	-	*n* = 15 74.69 ± 1.32 (64.00–83.25)	*n* = 15 86.68 ± 1.36 (78.00–98.50)	**<0.001**
Screening SBP (mmHg)	122.26 ± 1.71 (108.67–138.00)	150.46 ± 1.90 (139.00–167.33)	**<0.001**	123.41 ± 1.55 (112.00–139.00)	149.78 ± 2.16 (133.00–170.00)	**<0.001**
Screening DBP (mmHg)	78.62 ± 1.11 (68.33–86.67)	94.73 ± 2.14 (74.00–113.67)	**<0.001**	78.91 ± 1.54 (64.00–89.50)	78.91 ± 1.54 (64.00–89.50)	**<0.001**
sAA baseline (U/mL)	53.86 ± 8.20 (5.98–177.66)	34.22 ± 6.53 (2.17–97.87)	0.077	45.79 ± 9.15 (1.61–156.41)	80.34 ± 14.51 (2.11–206.29)	**0.047**
NE baseline (pg/mL)	326.33 ± 23.92 (166.40–533.20)	416.88 ± 26.79 (209.60–650.70)	**0.016**	*n* = 21 341.18 ± 32.98 (145.61–825.18)	507.61 ± 59.53 (170.21–1166.00)	**0.018**

Values are means ± standard error of the mean (range); NT = normotensive participants; HT = hypertensive participants; BMI = body mass index; BP = blood pressure; SBP = systolic blood pressure; DBP = diastolic blood pressure; sAA = salivary alpha-amylase; NE = norepinephrine; *n* = sample size; deviating sample sizes of a parameter are indicated; statistically significant results are highlighted in bold.

**Figure A1:**
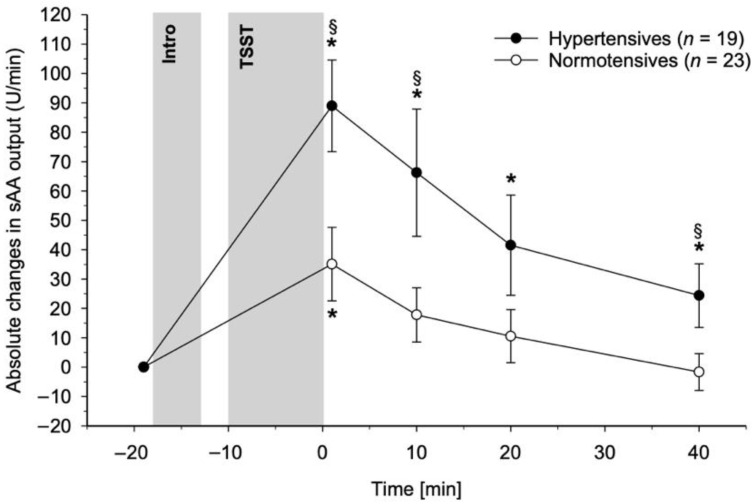
Salivary alpha-amylase (sAA) output reactivity, i.e., sAA reactivity with control for salivary flow rate, to the TSST in hypertensive individuals (black dots) and normotensive controls (white dots). Repeated measures ANCOVA revealed that hypertensive individuals showed higher sAA output reactivity to the TSST as compared to normotensive controls (*p* = 0.046; with covariates age and BMI: *p* = 0.096). Paragraphs (§) indicate significant differences between hypertensive and normotensive participants. Asterisks (*) indicate significant differences from baseline within the respective group.

## Data Availability

Data available on reasonable request from the authors.

## Appendix A

### Appendix A.1. Essential Hypertension Assessment

#### Appendix A.1.1. Stress Study

In this study, hypertension status was assessed by three seated screening blood pressure (BP) measurements obtained on three different days after 15 min rest using fully automated sphygmomanometry (Omron 773; Omron Healthcare Europe B.V., Hoofdorp, The Netherlands). One of the BP measurements was taken by the subject himself after careful instruction and training. The two further BP measurements were obtained by trained members of the study team. Average screening BP was computed, and participants were categorized into hypertensive (HT) and normotensive (NT) individuals based on the computed average screening BP following the World Health Organization (WHO)/International Society of Hypertension (ISH) definition (hypertension: SBP ≥ 140 mmHg and/or DBP ≥ 90 mmHg) [31]. Causes for secondary hypertension were excluded based on self-report in a preceding telephone interview.

#### Appendix A.1.2. Norepinephrine Infusion Study

For the assessment of hypertension status, we applied a two-step procedure comprising home and screening BP-measurements in this study. (1) Home BP measurements were obtained in a seated position after a minimum of 15 min rest twice per day (once in the morning and once in the evening) on up to three separate days using sphygmomanometry (Omron M6; Omron Healthcare Europe B.V., Hoofdorp, The Netherlands) by participants themselves. Average home BP was computed, and participants were preliminarily categorized as HT or NT following the European Society of Hypertension recommendations for home BP measurements (hypertension: average home SBP ≥ 135 mmHg and/or average home DBP ≥ 85 mmHg) [35]. (2) Preliminary home BP classification was extended by two additional screening BP measurements obtained by trained personnel using automated sphygmomanometry (Eagle 4000, Software Version 6F, Marquette Hellige GmbH, Freiburg) after resting periods of at least 5 min. For categorization in HT and NT, we applied the WHO/ISH definition (hypertension: SBP ≥ 140 mmHg and/or DBP ≥ 90 mmHg) [31]. This two-step procedure aimed, in combination with a comprehensive blood examination, at excluding secondary, white coat, and masked hypertension.

Notably, 7 NT and 5 HT did not provide home BP measurements. In order to nevertheless maintain a two-step procedure for the assessment of essential hypertension, we considered the mean of two resting study BP readings obtained on the second and third study day in these participants instead.

## Appendix B

### Appendix B.1. Salivary Flow Rate

To rule out confounding effects of salivary flow rate on our results [56] and thus to exclude that the observed salivary alpha-amylase (sAA) reactivity differences result from differences between HT and NT in salivary flow rate, we repeated our sAA analyses for the stress study controlling for salivary flow rate. The weight of the each salivette was assessed before and after 1 min of chewing. Flow rate was calculated as difference in salivette weight before and after chewing and expressed in g/min [60]. sAA output was then calculated by the formula sAA output (U/min) = sAA concentration (U/mL) × salivary flow rate (g/min) [55]. We first compared salivary flow rate between HT and NT at every measurement time point by calculating univariate analyses of variance (ANOVAs). Second, we repeated the sAA analyses for the stress study with sAA output instead of sAA concentrations.

#### Appendix B.1.1. Comparison of Salivary Flow Rate in Hypertensive and Normotensive Participants

There were no differences in salivary flow rate between HT and NT at any of the measurement time points (*p*’s ≥ 0.12).

#### Appendix B.1.2. Salivary Alpha-Amylase Reactivity to Acute Psychosocial Stress Controlling for salivary Flow Rate

HT exhibited higher sAA output reactivity, i.e., with control for salivary flow rate, to stress as compared to NT without (interaction group-by-time: *F*(2.83, 113.16) = 2.80, *p* = 0.046, η_p_^2^ = 0.07, *f* = 0.27) and after controlling for age and BMI as potential confounders (interaction group-by-time: *F*(3.01, 114.49) = 2.16, *p* = 0.096, η_p_^2^ = 0.05, *f* = 0.23; Figure A1). Similar to our sAA post hoc testing without consideration of salivary flow rate (see Section 3.2.1), comparison of sAA output level changes from baseline between HT and NT at each measurement time point revealed significantly higher sAA output increases in HT as compared to NT +1 min, +10 min, and +40 min after stress termination (*p*’s ≤ 0.036). No significant group differences were observed +20 min after stress termination (*p* = 0.10). Moreover, a repeat of the analyses performed separately for each group, while controlling for salivary flow rate, showed that both HT and NT exhibited a significant increase in sAA output in response to stress (main effect of time: NT: *F*(3.35, 73.71) = 4.39, *p* = 0.005, η_p_^2^ = 0.17, *f* = 0.45; HT: *F*(2.29, 41.29) = 10.04, *p* < 0.001, η_p_^2^ = 0.36, *f* = 0.75). NT showed significant increases in sAA output as compared to baseline + 1 min after stress (*p* = 0.010) and marginally increased sAA levels + 10 min after stress (*p* = 0.067) but not later (*p*’s ≥ 0.26) while in HT sAA output was elevated at all measurement time points after stress compared to baseline (*p*’s ≤ 0.038).
